# Physical exercise frequency and cognition: a multicenter cross-sectional cohort study

**DOI:** 10.3389/fnagi.2024.1381692

**Published:** 2024-03-08

**Authors:** Chen Wen, Jing-Huan Gan, Guo-Wei Huang, Xiao-Dan Wang, Yang Lü, Jian-Ping Niu, Xin-Ling Meng, Pan Cai, Yang Li, Bao-Zhi Gang, Yong You, Yan Lv, Zhi-Hong Ren, Shuai Liu, Yan Zeng, Yong Ji

**Affiliations:** ^1^Department of Neurology, Beijing Tiantan Hospital, Capital Medical University, China National Clinical Research Center for Neurological Diseases, Beijing, China; ^2^Department of Neurology, Beijing Friendship Hospital, Capital Medical University, Beijing, China; ^3^Department of Nutrition and Food Science, Tianjin Key Laboratory of Environment, Nutrition and Public Health, School of Public Health, Tianjin Medical University, Tianjin, China; ^4^Department of Neurology, Tianjin Huanhu Hospital, Tianjin Key Laboratory of Cerebrovascular and Neurodegenerative Diseases, Tianjin Dementia Institute, Tianjin, China; ^5^Department of Geriatrics, The First Affiliated Hospital of Chongqing Medical University, Chongqing, China; ^6^Department of Neurology, The Second Affiliated Hospital of Xiamen Medical College, Xiamen, China; ^7^Department of Neurology, Affiliated Traditional Chinese Medicine Hospital of Xinjiang Medical University, Urumqi, China; ^8^Dementia Clinic, Affiliated Hospital of Zunyi Medical University, Zunyi, China; ^9^Department of Neurology, The First Hospital of Shanxi Medical University, Taiyuan, China; ^10^Department of Neurology, The First Affiliated Hospital of Harbin Medical University, Harbin, China; ^11^Department of Neurology, Second Affiliated Hospital of Hainan Medical University, Haikou, China; ^12^Department of Neurology, Hainan General Hospital, Haikou, China; ^13^Department of Neurology, Beijing Electric Power Hospital, State Grid Corporation of China, Capital Medical University, Beijing, China; ^14^Brain Science and Advanced Technology Institute, Wuhan University of Science and Technology, Wuhan, China

**Keywords:** physical exercise frequency, dementia, cognitive impairment, Alzheimer’s disease, healthy guidance

## Abstract

**Background and aims:**

Dementia imposes a heavy burden on society and families, therefore, effective drug treatments, exploring and preventing factors associated with dementia, are paramount. To provide reference points for the best frequency of physical exercise (physical exercise), we investigated the association between frequency of PE and cognition in Chinese old adults.

**Methods:**

16,181 Chinese participants aged 65 years or older were included in this study. Associations between PE and cognition were estimated multivariate logistic and linear regression analyses. Associations were further investigated across dementia subtypes (Alzheimer dementia, vascular dementia, and other types of dementia). Subgroup analyses were performed in different age groups, in populations with and without stroke, and those with and without hypertension.

**Results:**

PE associated with dementia after adjusting for full covariates (OR: 0.5414, 95% CI: 0.4536–0.6491, *p* < 0.001). Exercise performed at ≥3 times/week associated with lower risk of dementia (OR: 0.4794–0.6619, all *p* value <0.001). PE was associated with improved cognition (*β*: 12851, *p* < 0.001), and any PE frequency contributed to cognitive improvement (*p* values for exercise performed ≥1 time/week were <0.001). Similar conclusions were identified when we repeated analyses in different dementia subtypes and age groups. Subgroup analyses suggested that the cognition of individuals without hypertension also benefitted from exercising 1–2 times/week (OR: 0.6168, 95% CI: 0.4379–0.8668, *p* = 0.005).

**Conclusion:**

The best exercise frequency is exercising ≥3 times/week for individuals from different dementia subtypes and age groups. While for those without hypertension, PE at 1–2 times /week is also beneficial.

## Introduction

Dementia leads to a loss of independence thereby affecting families and the economy. In global terms, China has the largest population of individuals with dementia (Jia et al., 2020). In populations aged ≥65 years, the prevalence of all-cause dementia is 9.11%, while this prevalence is higher in rural areas when compared with urban areas (Hu et al., 2022). In 2050, the annual total cost of dementia will be approximately $1.89 trillion (Jia et al., 2020). Currently, no disease-modifying treatments are available for dementia. Therefore, exploring dementia prevention mechanisms and risk reduction approaches is paramount in China (Gao and Jia, 2023). Previous studies have shown that physical exercise (PE) is a potential cognition protective factor for individuals in early dementia stages (Liu et al., 2022), such as subjective cognitive decline (Wen et al., 2021) and mild cognitive impairment (Law et al., 2020). These findings suggest that PE interventions can affect cognition at an earlier stage than previously thought.

Physical inactivity is a modifiable risk factor associated with the reduced age-specific incidence of dementia (Tarassova et al., 2020; Alshagrawi and Abidi, 2023). Data from 7,000 individuals over a 2 years follow-up period showed that PE prevented or delayed cognitive impairment progression (He et al., 2021). PE increases cerebral blood flow (CBF) and nervous system plasticity (Farì and Lunetti, 2021). PE also reduces the neuroinflammation, oxidative stress, and amyloid β-protein (Aβ) deposition (Zhang et al., 2019). However, some studies have also shown inconsistent results; after a 5 years exercise intervention, older individuals showed no significant improvements in cognition (Zotcheva et al., 2022). Meanwhile, the most effective PE modalities for different population subgroups remain limited (Bull et al., 2020). Thus, there is a need for large sample studies to confirm such associations and provide evidence for the best PE intervention modality (Cha, 2022). To address this, we assessed the effects of PE on dementia and provided evidence showing the best PE interventions in China.

## Materials and methods

### Participants

This study is our second multicenter, cross-sectional epidemiological survey, from April to October 2019, of dementia in elderly Chinese participants aged 65 years or older.

**Figure 1:**
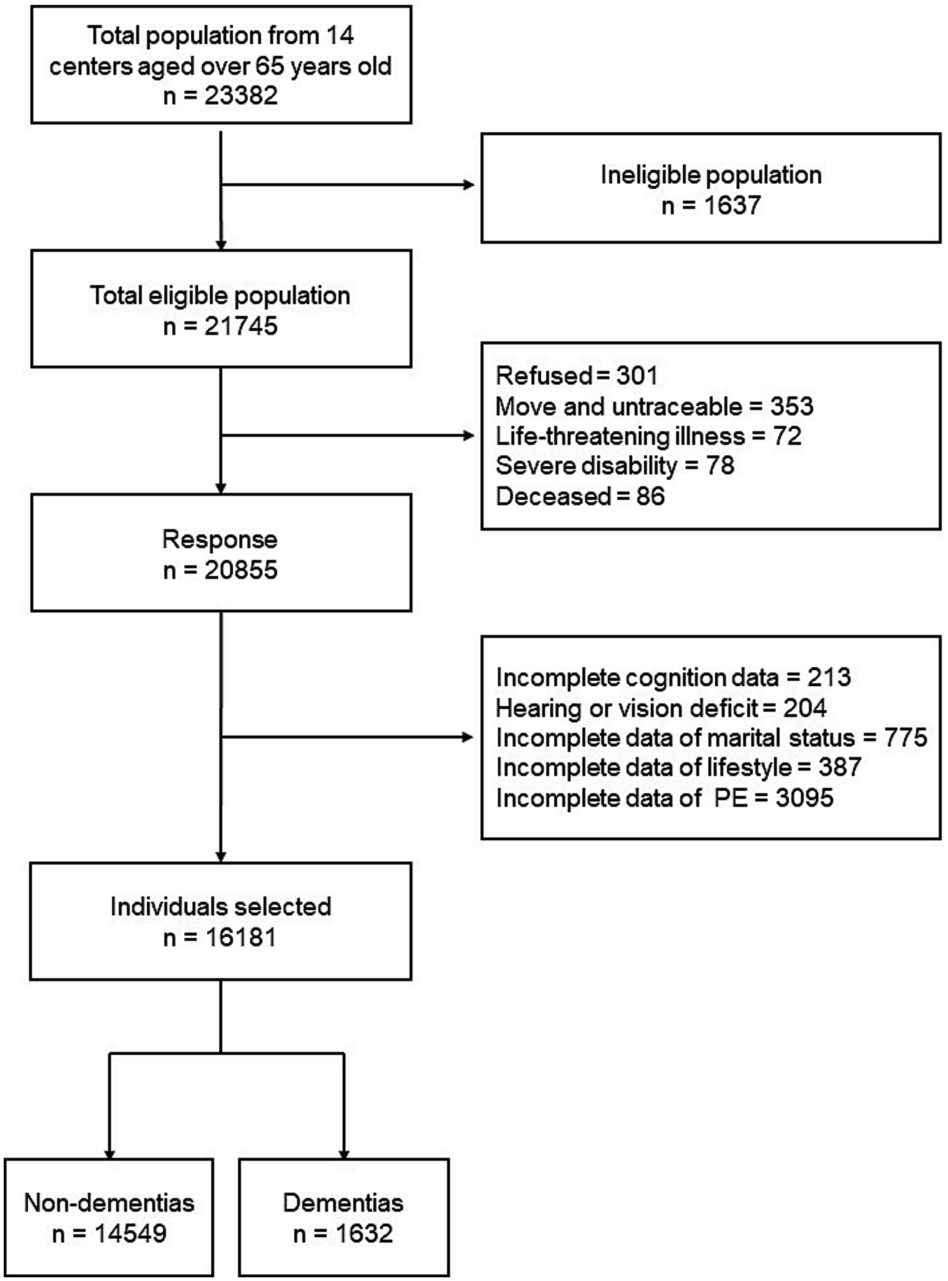
Flowchart.

We collected data from 13 provinces, metropolitan areas, and autonomous areas which represented different geographical regions, urbanization levels, and economic development status in China. These areas included: Beijing, Tianjin, Chongqing, Fujian, Guizhou, Heilongjiang, Hubei, Hebei, Henan, Hunan, Liaoning, Shanxi, and Xinjiang. The detailed multistage, stratified cluster-sampling procedure was described in our previous study (Chen et al., 2022). A total of 23,382 individuals were interviewed, while only 21,745 individuals were eligible. All eligible individuals were aged 65 years or older and had lived in the same community or village for at least 1 year preceding the survey date. Among the eligible population, 301 refused to participate; 353 were untraceable; 72 had life-threatening illness; 78 were severe disability; 86 were deceased. Therefore, information from 20,855 individuals were collected. After excluding individuals with incomplete data and hearing or vision deficit, we included 16,181 individuals in our study (Figure 1).

### Screening interview

This cross-sectional, door-to-door, questionnaire-based survey was conducted by senior neurologists and medical staff. All interviewers and experts received the same training on collecting information, neuropsychological assessments, and diagnosis, and retrained every 2 months. Participants’ findings were recorded from physical and neurological examinations.

During interviews, participants completed a self-designed questionnaire using help from reliable informants (participant’s spouse, children, other relatives or close friends, in descending order). Informants provided information if participants were unable to do so. The average interview lasted 30 min. Information collected from questionnaire included demographic factors (age, sex, and education years), lifestyle factors (smoking, drinking, PE status and PE frequency), and comorbidities (hypertension, diabetes, cerebrovascular disease etc). PE was defined as performing physical exercise that lasted 30 min or more and was evaluated with reference to a health survey (Kurtze et al., 2008). Participants were thought to perform PE if they answered “yes” for the question “Do you perform physical exercise that lasted 30 min or more?” PE frequency was collected by asking “How often do you perform physical exercise?” The answer was selected from the following choice:: 0 times/week (never), 1–2 times/week, 3 times/week, 4–5 times/week, and >5 times/week. The answers would be confirmed by reliable informants.

Comorbidities, including stroke, hypertension, diabetes mellitus (DM), and coronary heart disease (CHD) history, were recorded from medical registers, and then confirmed with senior neurologists and medical staff to ensure accuracy. Stroke was defined as having a diagnosed or a known history of hemorrhagic or ischemic stroke. Hypertension was defined as having an average systolic blood pressure ≥ 140 mmHg or an average diastolic blood pressure ≥ 90 mmHg on ≥ three occasions or patients taking antihypertensive drugs. DM was defined as having a fasting serum glucose level ≥ 7 mmol/L, a non-fasting serum glucose level ≥ 11.1 mmol/L, or using hypoglycemic agents. CHD was defined as coronary atherosclerotic heart disease, which meant heart disease caused by coronary artery stenosis or occlusion.

### Cognitive evaluation and dementia criteria

The Chinese Mini-Mental State Examination (C-MMSE) (Arevalo-Rodriguez et al., 2015), the Clinical Dementia Rating (CDR) scale (Morris, 1993), and Activities of Daily Living (ADL) scale (Eto et al., 1992; Chen et al., 1995) were administered by qualified and experienced specialists in neurology. Interviewers at each site included four junior neurologists and four neurologists from the local cooperative hospital. An expert panel and interviewers reviewed all the gathered information, and primary diagnoses were made at the end of each workday. If consensus was not reached, an expert returned to the participant’s residence the following day to reexamine and reevaluate the participant and provide a final, definitive diagnosis. Data were stored on a secure server accessible by authorized personnel only.

In our survey, a non-dementia status was assigned when participants scored 0 on global CDR and ≥27 on the C-MMSE. When the C-MMSE test score was ≤ the cutoff point (≤17 for illiterate persons, ≤20 for persons with 1–6 years of education, and ≤24 for persons with ≥7 years of education), dementia was defined based on clinical criteria from the Diagnostic and Statistical Manual of Mental Disorders (DSM-IV edition). DSM-IV criteria for dementia required an impairment in memory and at least one additional cognitive domain; an impairment that resulted in a significant decline from a previous functioning, a gradual onset and progressive course, and not due to any other process. An Alzheimer’s disease (AD) diagnosis was based on The National Institute of Neurological and Communicative Disorders-AD and Related Disorders Association criteria (McKhann et al., 2011). We used diagnostic criteria for vascular dementia (VD) as proposed by the Neuroepidemiology Branch of the National Institute of Neurological Disorders and Stroke (Román et al., 1993). Other types of dementia (mixed dementia, frontotemporal dementia, dementia with Lewy bodies, Parkinson’s disease, alcoholic dementia, hydrocephalus dementia, and posttraumatic dementia) were defined by globally accepted criteria (McKeith et al., 2017).

### Ethical considerations

The study was approved by the ethics committee of Tianjin Huanhu Hospital (ID:2019-40). Written informed consent was obtained from participants or their guardians. The procedures were performed in accordance with the ethical standards of the Committee on Human Experimentation. Study data were anonymous.

### Statistical analysis

We used Kolmogorov–Smirnov normality tests and Quantile-Quantile plots to assess data normality. Variables were transformed using the “car” package in R software (Xu et al., 2020) to generate approximate normal distributions (Supplementary Figure S1). Statistical analyses were conducted on transformed values. Differences in categorical variables between two groups were analyzed using Chi-square tests, and numerical variables were analyzed using Wilcoxon tests. Difference comparisons between two groups involved multiple comparisons, which may have generated uncontrolled type I error rates (the rate of rejecting the null hypothesis when it should not be rejected) (Cabral, 2008). We used the false discovery rate (FDR) to adjust for multiple comparisons (threshold *q* < 0.05).

### Model building and covariate selection

Statistically significant indicators in univariate analysis (age, sex, education years, and stroke, Supplementary Tables S1, S2) were included in multivariate analysis. Although no significance was identified for hypertension, DM, CHD, smoking, and drinking, they were previously considered risk factors for dementia (Baumgart et al., 2015) and were also included in multivariate analysis and categorized. M2 was adjusted for demographic factors (age, sex, and education years). M3 was additionally adjusted for comorbidities and lifestyle indicators (stroke, hypertension, DM, CHD, smoking, and drinking).

First, associations between PE (status and frequency) and dementia were estimated using a univariate logistic regression model (M1). Then, associations were further confirmed using a multivariate logistic regression model (M2). Finally, indicators were added to the multivariate logistic regression model as covariates to assess the robustness of results (M3).

Considering the fact that PE could affect cognition, we investigated if PE (status and frequency) was associated with C-MMSE scores. Associations between PE and cognition were estimated using linear regression models M1–M3, which adjusted for the same aforementioned factors. We also conducted an association study in different dementia subtypes, in participants with AD, VD, and other dementia types.

Variables in multivariate regression analyses were selected for interaction analyses. Accordingly, we conducted subgroup analyses stratified by age (65–74, or ≥75), hypertension (yes or no), and stroke (yes or no). Variance inflation factors were used to assess multicollinearity, which we found no evidence of in our analyses.

Two-tailed *p* < 0.05 values were considered statistically significant. Analyses were conducted in R software (version 3.6.1).

## Results

### Participant characteristics

Participant characteristics are shown (Table 1). In total, 16,181 participants (14,549 with non-dementia and 1,632 with dementia) were included. In the population, the average age was 74.35 years (±6.31 years) and the average education duration was 7.07 years (±4.73 years). We used FDR values (*q* values) to adjust false positive results in multiple comparisons. When compared with non-dementia, participants with dementia were older (73.92 ± 6.03 vs. 78.11 ± 7.389, *q* value <0.001), less educated (education years 7.21 ± 4.75 vs. 5.89 ± 4.43, *q* value <0.001), had a larger percentage of females (55.62% vs. 62.81%, *q* value <0.001), a larger percentage of stroke (13.29% vs. 18.08%, *q* value <0.001) and worse cognitive performance (C-MMSE score = 26.90 ± 3.17 vs. 15.93 ± 4.93, *q* value <0.001). Participants with dementia performed less PE and had lower PE frequencies (*q* value <0.001). No significant differences were identified for hypertension, DM, CHD, smoking, and drinking (*q* value >0.05).

**Table 1:** Characteristic of participants.

Characteristics	Non-dementia (*n* = 14,549)	Dementia (*n* = 1,632)	Total (*n* = 16,181)	*p* value	*q* value
Age (years)	73.92 ± 6.03	78.11 ± 7.389	74.35 ± 6.31	<0.001	<0.001
Sex (female)	8,092 (55.62%)	1,025 (62.81%)	9,117 (56.34%)	<0.001	<0.001
Education (years)	7.21 ± 4.75	5.89 ± 4.43	7.07 ± 4.73	<0.001	<0.001
C-MMSE score	26.90 ± 3.17	15.93 ± 4.93	25.79 ± 4.74	<0.001	<0.001
Hypertension (yes)	7,263 (49.92%)	816 (50.00%)	8,079 (49.93%)	0.973	0.990
DM (yes)	2,184 (15.01%)	239 (14.64%)	2,423 (14.97%)	0.721	0.865
CHD (yes)	2,129 (14.63%)	250 (15.32%)	2,379 (14.70%)	0.481	0.642
Stroke (yes)	1,933 (13.29%)	295 (18.08%)	2,228 (13.77%)	<0.001	<0.001
Smoking (yes)	3,781 (25.99%)	451 (27.63%)	4,232 (26.15%)	0.160	0.240
Drinking (yes)	3,354 (23.05%)	377 (23.10%)	3,731 (23.06%)	0.990	0.990
Physical exercise (yes)	13,784 (94.74%)	1,442 (88.36%)	15,226 (94.10%)	<0.001	<0.001
Frequency of physical exercise				<0.001	<0.001
Never	765 (5.26%)	190 (11.64%)	955 (5.90%)		
1–2 times/week	933 (6.41%)	170 (10.42%)	1,103 (6.82%)		
3 times/week	1,649 (11.33%)	229 (14.03%)	1,878(11.61%)		
4–5 times/week	6,342 (43.59%)	587 (35.97%)	6,929(42.82%)		
>5 times/week	4,860 (33.40%)	456 (27.94%)	5,316(32.85%)		

Continuous variables were represented as mean ± SD. Categorical variables were represented as numbers (proportion). Differences in categorical variables between two groups were analyzed using Chi-square tests, and numerical variables were analyzed using Wilcoxon tests. *q* value: significance after false discovery rate (FDR) correction. C-MMSE, Chinese Mini-Mental State Examination; DM, diabetes mellitus; CHD, coronary heart disease.

### Associations between physical exercise and dementia

**Figure 2:**
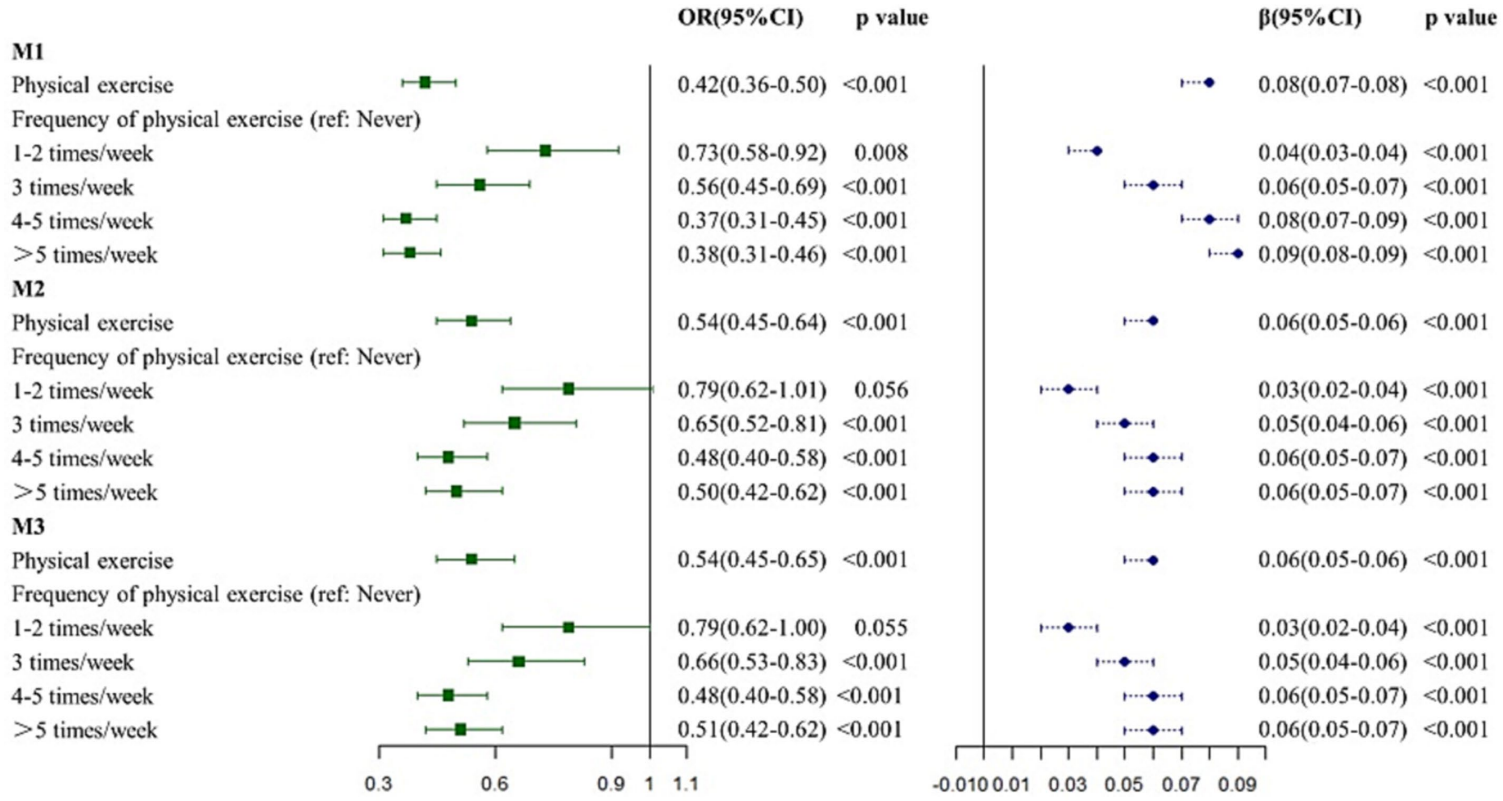
Association between physical exercise and dementia. M1: the univariate regression analysis. M2: the multivariate analysis adjusted for age, sex, and education years. M3: the multivariate analysis adjusted for age, sex and education years, hypertension, DM, CHD, stroke, smoking and drinking.

From univariate logistic regression analysis (Figure 2 and Supplementary Table S3), PE was associated with dementia (odds ratio (OR): 0.4212, 95% confidence interval (CI): 0.3569–0.4994, *p* < 0.001) regardless of the frequency (1–2 times/week: OR: 0.7336, 95% CI: 0.5838–0.9212, *p* = 0.008; 3 times/week: OR: 0.5591, 95% CI: 0.4531–0.6906, *p* < 0.001; 4–5 times/week: OR: 0.3727, 95% CI: 0.3118–0.4470, *p* < 0.001; and >5 times/week: OR: 0.3778, 95% CI: 0.3142–0.4556, *p* < 0.001). After adjusting for age, sex, and education duration (years) in M2, PE appeared to protect participants from dementia (OR: 0.5379, 95% CI: 0.4512–0.6441, *p* < 0.001), especially for PE at ≥3 times/week (3 times/week: OR: 0.6497, 95% CI: 0.5217–0.8099, *p* < 0.001; 4–5 times/week: OR: 0.4772, 95% CI: 0.3956–0.5778, *p* < 0.001; and >5 times/week: OR: 0.5044, 95% CI: 0.4150–0.6150, *p* < 0.001). However, performing PE 1–2 times/week did not make any difference (OR: 0.7927, 95% CI: 0.6244–1.0059, *p* = 0.056). In M3 the association between PE and dementia remained significant (OR: 0.5414, 95% CI: 0.4536–0.6491, *p* < 0.001). When compared with inactivity, performing PE ≥ 3 times/week was a protective factor for dementia (3 times/week: OR: 0.6619, 95% CI: 0.5310–0.8259, *p* < 0.001; 4–5 times/week: OR: 0.4794, 95% CI: 0.3969–0.5811, *p* < 0.001; and >5 times/week: OR: 0.5053, 95% CI: 0.4152–0.6170, *p* < 0.001).

### Association between physical exercise and cognition

PE was positively associated with cognition in unadjusted model (*β*: 26944, *p* < 0.001, Figure 1 and Supplementary Table S4). The association remained significant after adjusting for age, sex, and education years (M2, *β*: 13013, *p* < 0.001) and full covariate adjustment (M3, *β*: 12851, *p* < 0.001). In terms of PE frequency, performing PE ≥ 1 time/week had a positive effect on cognition in univariate linear regression analysis M1 (1–2 times/week: *β*: 9259, *p* < 0.001; 3 times/week: *β*: 18463, *p* < 0.001; 4–5 times/week: *β*: 26858, *p* < 0.001; and >5 times/week: *β*: 33721, *p* < 0.001). Consistent with M1, performing PE ≥ 1 time/week was positively associated with cognition in M2 (1–2 times/week: *β*: 6675, *p* < 0.001; 3 times/week: *β*: 12823, *p* < 0.001; 4–5 times/week: *β*: 13395, *p* < 0.001; and >5 times/week: *β*: 14222, *p* < 0.001), and M3 (1–2 times/week: *β*: 6788, *p* < 0.001; 3 times/week: *β*: 12648, *p* < 0.001; 4–5 times/week: *β*: 13221, *p* < 0.001; and >5 times/week: *β*: 14036, *p* < 0.001).

### Association between physical exercise and dementia subtype

**Figure 3:**
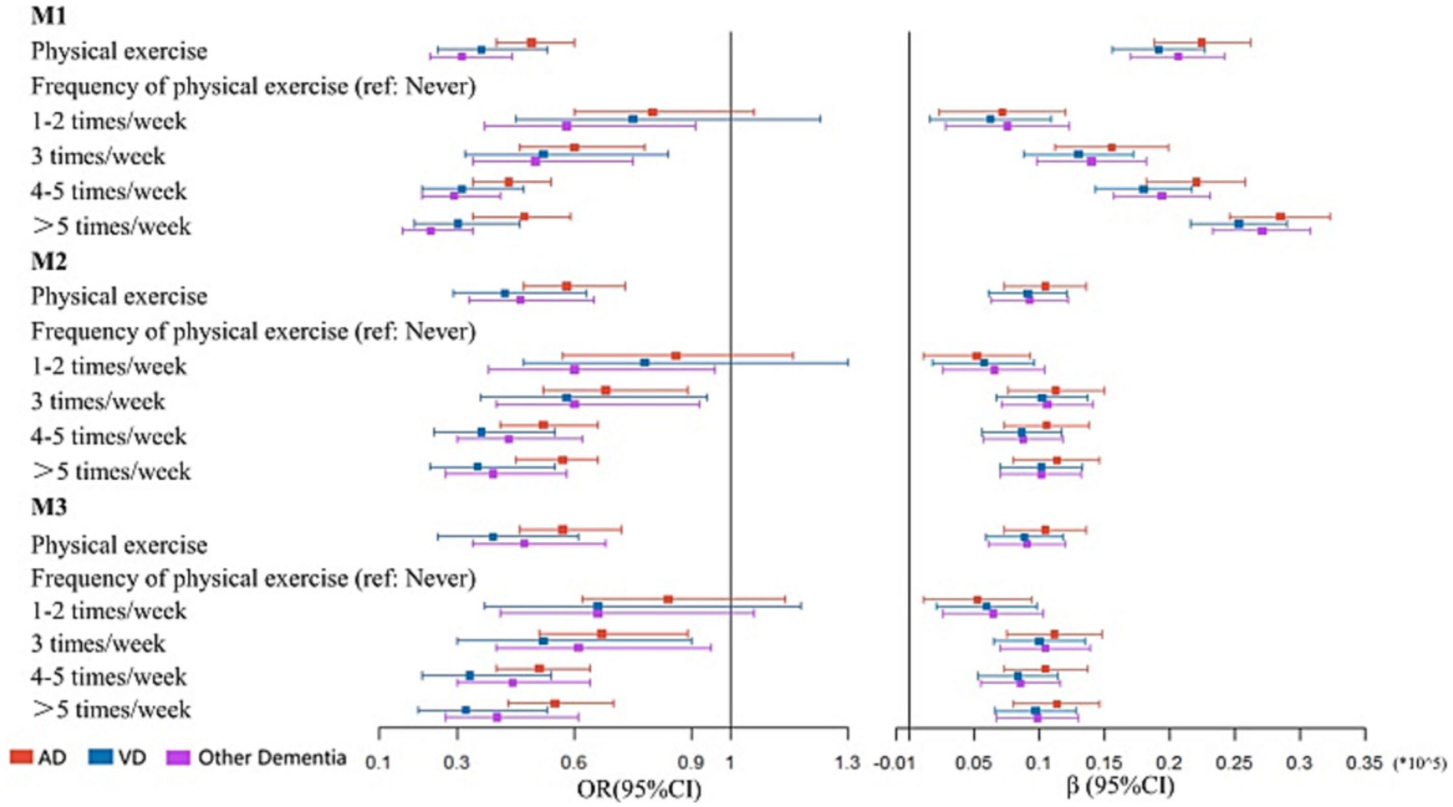
Association between physical exercise and dementia subtype. M1: the univariate regression analysis. M2: the multivariate analysis adjusted for age, sex, and education years. M3: the multivariate analysis adjusted for age, sex and education years, hypertension, DM, CHD, stroke, smoking and drinking.

The results for different dementia subtypes were largely similar to the above analyses (Figure 3 and Supplementary Table S5). PE (M3: OR: 0.5708, 95% CI: 0.4578–0.7178, *p* < 0.001) and performing PE ≥ 3 times/week (M3: 3 times/week: OR: 0.6741, 95% CI: 0.5115–0.8906, *p* = 0.005; 4–5 times/week: OR: 0.5055, 95% CI: 0.3996–0.6439, *p* < 0.001; and >5 times/week: OR: 0.5450, 95% CI: 0.4276–0.6992, *p* < 0.001) was associated with AD, while no association was observed between AD and performing PE 1–2 times/week (*p* = 0.259). When compared with inactivity, PE (M3: *β*: 10406, *p* < 0.001) and performing PE ≥ 1 time/week (M3: 1–2 times/week: *β*: 5219, *p* = 0.013; 3 times/week: *β*: 11134, *p* < 0.001; 4–5 times/week: *β*: 10464, *p* < 0.001; and >5 times/week: *β*: 11325, *p* < 0.001) improved cognition in AD participants.

VD was associated with PE (M3: OR: 0.3864, 95% CI: 0.2536–0.6052, *p* < 0.001). Performing PE ≥ 3 times/week indicated the most beneficial frequency for VD (M3: 3 times/week: OR: 0.5216, 95% CI: 0.3044–0.8981, *p* = 0.018; 4–5 times/week: OR: 0.3328, 95% CI: 0.2101–0.5383, *p* < 0.001; and >5 times/week: OR: 0.3239, 95% CI: 0.1994–0.5346, *p* < 0.001). Cognitive improvement in VD participants was associated with PE (M3: *β*: 8806, *p* < 0.001) and performing PE ≥ 1 time/week (M3: 1–2 times/week: *β*: 5907, *p* = 0.003; 3 times/week: *β*: 9987, *p* < 0.001; 4–5 times/week: *β*: 8334, *p* < 0.001; and >5 times/week: *β*: 9694, *p* < 0.001).

PE (M3: OR: 0.4749, 95% CI: 0.2536–0.5295, *p* < 0.001) and performing PE ≥ 3 times/week (M3: 3 times/week: OR: 0.6148, 95% CI: 0.4004–0.9462, *p* = 0.026; 4–5 times/week: OR: 0.4361, 95% CI: 0.3029–0.6369, *p* < 0.001; and >5 times/week: OR: 0.4033, 95% CI: 0.2707–0.6057, *p* < 0.001) appeared to associated with lower risk of other dementia types. Consistent with aforementioned analyses, PE (M3: *β*: 9043, *p* = 0.001) and performing PE ≥ 1 time/week (M3: 1–2 times/week: *β*: 6407, *p* < 0.001; 3 times/week: *β*: 10458, *p* < 0.001; 4–5 times/week: *β*: 8525, *p* < 0.001; and >5 times/week: *β*: 9839, *p* < 0.001, Supplementary Table S6) had positive effects on cognitive improvement in individuals with other dementia types. No multicollinearity was identified in our analyses (Supplementary Table S7).

### Subgroup analyses

Interaction analyses demonstrated that associations between PE frequency and dementia were possibly affected by age, hypertension status, and stroke status (Supplementary Table S8). Therefore, subgroup analyses were conducted at different ages (65–74, and ≥75 years, Supplementary Table S9), stroke status (yes and no, Supplementary Table S10) and hypertension status (yes and no, Supplementary Table S11).

**Figure 4:**
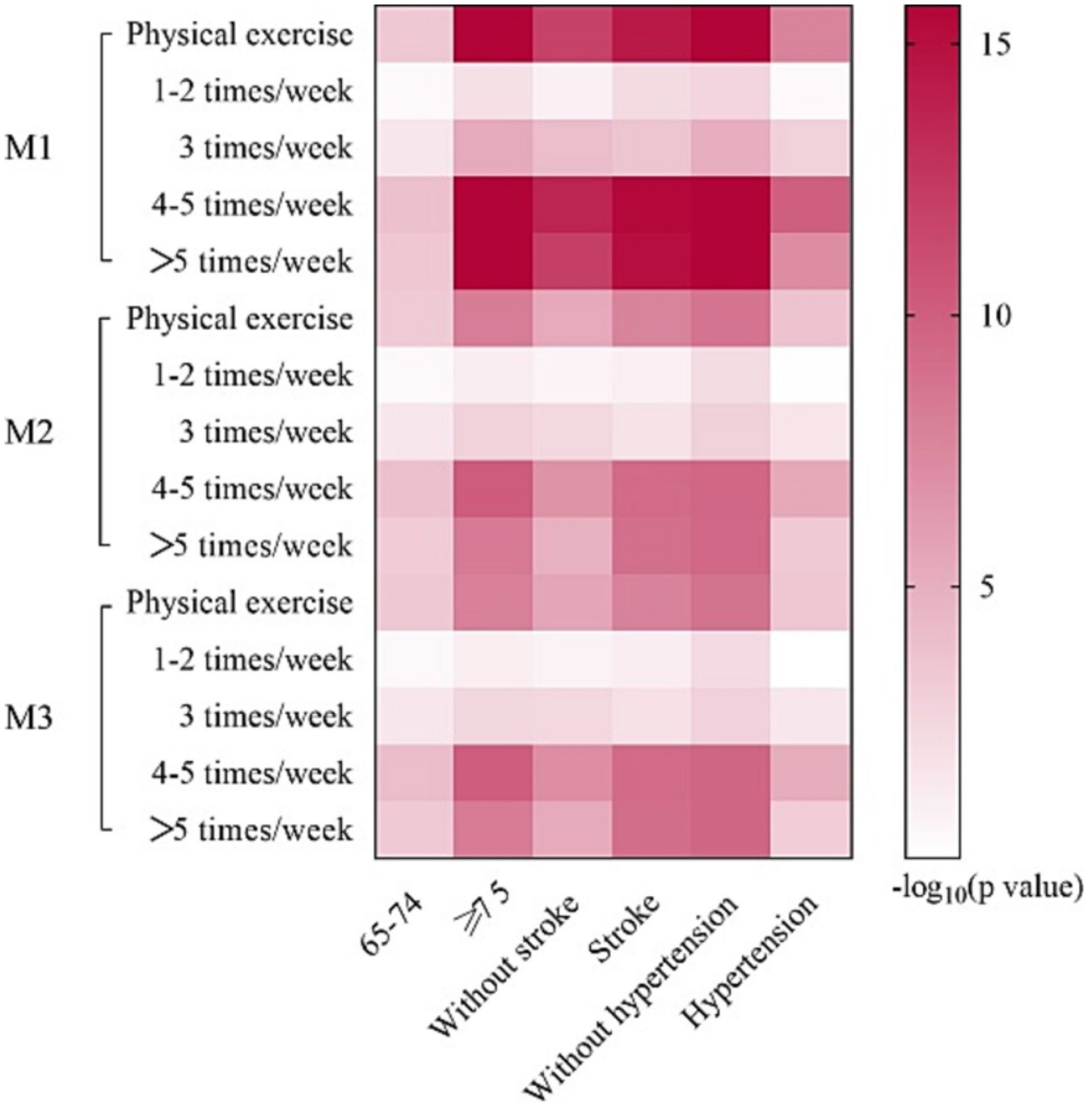
Logistic regression analyses of subgroup. M1: the univariate logistic regression analysis. M2: the multivariate logistic regression analysis adjusted for age, sex and education years. M3: the multivariate logistic regression analysis adjusted for age, sex and education years, hypertension, DM, CHD, stroke, smoking and drinking.

Subgroup analysis results were largely consistent with aforementioned analyses (Figure 4). However, some inconsistencies were identified. Participants without hypertension appeared to benefit from performing PE 1–2 times/week (M1: OR: 0.6094, 95% CI: 0.4404–0.8409, *p* = 0.003). This association remained significant in M2 (OR: 0.6255, 95% CI: 0.4446–0.8782, *p* = 0.007) and M3 (OR: 0.6168, 95% CI: 0.4379–0.8668, *p* = 0.005).

## Discussion

In a large sample cohort from China, we assessed associations between PE frequency and dementia. PE exerted positive effects on cognition in our cohort. When compared with inactivity and having a lower PE frequency (1–2 times/week), performing PE ≥ 3 times/week was better for improving cognition. Further findings, based on stratified analyses by age, hypertension, and stroke status, consistently and significantly showed the protective role of PE and performing PE ≥ 3 times/week. Of note, participants without hypertension might benefit from performing PE 1–2 times/week. Our findings strengthen the evidence showing the protective effects of PE frequency on dementia, and provide insights on PE for dementia prevention.

In our study, PE was a protective factor for dementia, regardless of age and comorbidity. These observations were consistent with previous studies; in a 10 years follow-up study, PE was inversely associated with cognitive impairment onset (Jedrziewski et al., 2010). Similarly, a systematic review involving 5,606 individuals from 73 articles concluded that all types of PE protected individuals from decreased global cognition (Huang et al., 2022). However, recent studies have also reported inconsistent results. A multicenter trial randomized 494 individuals, followed them for 4 months, and showed that moderate to high-intensity PE did not slow cognitive impairment in individuals with mild to moderate dementia (Lamb et al., 2018). A recent systematic review reported that neither a combination of strength and aerobic exercise, nor aerobic exercise alone, exerted beneficial effects toward cognition and dementia (Steichele et al., 2022). PE exposure and sample size heterogeneity could account for some of these inconsistencies. Other covariates potentially affecting cognition, such as lifestyle factors and comorbidity, may also contribute to this heterogeneity.

Different PE levels can generate different effects, but few studies have quantified PE interventions for dementia (López-Ortiz et al., 2021; Liu et al., 2022). Previous studies also confirmed a dose–response relationship between exercise and cognition (Gallardo-Gómez et al., 2022). Higher PE frequency appeared to contribute to better cognitive results, while specific PE thresholds require clarification (Jia et al., 2019). Our analyses indicated that performing PE ≥ 3 times/week was associated with dementia. Of note, performing PE 1–2 times/week was a protective factor for dementia in individuals without hypertension. This finding supported a previous study showing that low-frequency PE exerted positive effects on cognitive function in individuals with chronic diseases (Cai et al., 2017).

Although recommendations for PE levels and types for particular groups have been reported, the evidence for the best PE modality for individuals with different cognitive states remains uncertain (Ding et al., 2020; Steichele et al., 2022). In our study, we analyzed different dementia types, including AD, VD, and other dementias. Consistent with previous studies, we confirmed that PE was a protective factor against AD and benefited cognition (Norton et al., 2014). Regular PE protects non-dementia individuals from AD pathological changes in cerebrospinal fluid (CSF) (Zhong et al., 2022). Also, PE was associated with VD in our study. This finding supports previous studies showing that PE exerted beneficial effects toward VD and putatively prevented VD development (Aarsland et al., 2010). This association may relate to the fact that PE increases oligodendrocyte precursor cell populations in the sub ventricular zone of the brain (Ohtomo et al., 2020).

Animal model and human studies have explored underlying mechanisms at multiple levels (Stillman et al., 2020; Tarassova et al., 2020). Firstly, PE may increase cerebral perfusion by increasing cerebral blood flow (CBF) (Huang et al., 2022). Such increases during PE could meet the energy demands related to cognition in the brain and improve cognitive function (Buxton, 2021; Yamada et al., 2021). Results of the researches on the association between PE and cognition were inconsistent. Some studies have pointed out that high-intensity exercises were associated with hyperventilation and hypoxia, which constricted blood vessels and reduced CBF (Verges et al., 2012). This observation may explain a decline in cognition after high-intensity PE (Gallardo-Gómez et al., 2022). However, a study has pointed out that cognitive impairment caused by high-intensity exercise was not related to CBF (Komiyama et al., 2020). Our study explored the relationship between PE frequency and cognition in a large sample size. Secondly, PE improved cognition by promoting neuroplasticity and neuroprotection (Soshi et al., 1991; Farì and Lunetti, 2021). These processes were directly mediated by increased brain-derived neurotrophic factor (BDNF) levels induced by PE (Wheeler et al., 2020). BDNF activates multiple intracellular signaling pathways, including phospholipase C-γ1/protein kinase C, Ras-mitogen-activated protein kinases, and phosphoinositide 3-kinase/seronine protein kinase, to regulate cerebral cortex thickness and synaptic density, thus increasing brain plasticity (Wang and Holsinger, 2018). At peripheral levels, PE promoted fibronectin type III domain-containing 5 cleavage into irisin, which may have activated the brain cyclic adenosine phosphate/Protein Kinase A/cAMP-response element binding protein and enhanced BDNF levels (Madhu et al., 2022). PE also stimulated the ketone body D-β-hydroxybutyrate and cathepsin B to activate BDNF expression. Also, serotonin (Pietrelli et al., 2018) and several growth factors, including insulin growth factor-1 and vascular endothelial growth factor, were also induced by PE and exerted synergistic effects with BDNF in terms of neuroplasticity and neuroprotection (Jachim et al., 2020). Finally, neuroinflammation increases with aging and contributes to cognition decline, whereas PE was shown to attenuate this process (Huang et al., 2021). PE appeared to regulate micro-RNA expression (Hu et al., 2015), and significantly decreased pro-inflammatory markers, including interleukin-1β (IL-1β), IL-6, and tumor necrosis factor-α (TNF-α) (Qin et al., 2022). Proinflammatory microglia and astrocytes were suppressed by PE (Nakanishi et al., 2021). Additionally, PE was positively associated with CSF Aβ42 levels (Zhong et al., 2022), which may be mediated by activating lysosomal function (Wang et al., 2022) and promoting microglial Aβ clearance (Liang et al., 2022).

Our subgroup analysis showed that performing PE 1–2 times/week was associated with a lower risk of dementia in patients without hypertension. In participants with hypertension, performing PE ≥3 times/week was beneficial. Chronic hypertension had detrimental effects on cognition via mechanisms underpinning cerebral small vessel disease, reduced white matter integrity, and impaired autoregulation in the brain (Claassen et al., 2021; Triposkiadis and Xanthopoulos, 2023). Thus, hypertension may reduce or negate the benefits of low-frequency PE.

A major advantage of our study is its large sample size which provides considerable data reliability and robustness. Also, cognition-related covariate adjustments highlighted the independence of PE as a protective factor for dementia and cognition. Our dementia subtype analyses could help us understand the effects of PE on different dementia types. However, our study had notable limitations.

As a cross-sectional study, we did not provide causation information similar to other prospective cohorts. Objective measurements for PE, such as pulse oximetry or the calculation of “mets,” and some PE-related indicators (types, intensity, or duration) were unavailable. Information about the consistent length of comorbidities was not collected. Additionally, participants were primarily elderly Chinese individuals, thus diversity across ages, races, and regions was not confirmed, and so populations should be expanded to identify more generalizable findings. Finally, our study was limited to dementia population and cognitively normal population. More studies should be performed in individuals at pre-clinical dementia stages so appropriate PE interventions can be implemented for these individuals.

## Conclusion

In conclusion, PE was associated with cognitive decline when different adjustments were applied, regardless of dementia subtype. Performing PE ≥ 3 times/week was most effective in preventing dementia, whereas cognition appeared to benefit from any PE frequency. Moreover, the protective effects of PE were consistently observed in subgroups. Our findings underscore the importance of PE as a non-pharmaceutical therapy for delaying cognitive decline and preventing dementia in China.

## Data availability statement

The raw data supporting the conclusions of this article will be made available by the authors, without undue reservation.

## Ethics statement

The studies involving humans were approved by the ethics committee of Tianjin Huanhu Hospital. The studies were conducted in accordance with the local legislation and institutional requirements. The participants provided their written informed consent to participate in this study.

## Author contributions

CW: Formal analysis, Investigation, Software, Writing – original draft, Writing – review & editing. J-HG: Formal analysis, Investigation, Writing – review & editing. G-WH: Investigation, Resources, Writing – review & editing. X-DW: Investigation, Writing – review & editing. YLü: Investigation, Validation, Writing – review & editing. J-PN: Investigation, Visualization, Writing – review & editing. X-LM: Investigation, Writing – review & editing. PC: Investigation, Writing – review & editing. YLi: Investigation, Writing – review & editing. B-ZG: Investigation, Writing – review & editing. YY: Investigation, Writing – review & editing. YLv: Investigation, Writing – review & editing. Z-HR: Investigation, Writing – review & editing. SL: Data curation, Investigation, Supervision, Writing – review & editing. YZ: Investigation, Resources, Visualization, Writing – review & editing. YJ: Conceptualization, Funding acquisition, Investigation, Methodology, Project administration, Resources, Validation, Writing – review & editing.

## References

[ref1] Aarsland D. Sardahaee F. S. Anderssen S. Ballard C. (2010). Is physical activity a potential preventive factor for vascular dementia? A systematic review. Aging Ment. Health 14, 386–395. doi: 10.1080/13607860903586136, PMID: 20455113

[ref2] Alshagrawi S. Abidi S. T. (2023). Efficacy of an mHealth behavior change intervention for promoting physical activity in the workplace: randomized controlled trial. J. Med. Internet Res. 25:e44108. doi: 10.2196/44108, PMID: 37103981 PMC10176147

[ref3] Arevalo-Rodriguez I. Smailagic N. Roqué I. F. M. Ciapponi A. Sanchez-Perez E. Giannakou A.. (2015 (2015) Cd010783). Mini-mental state examination (MMSE) for the detection of Alzheimer's disease and other dementias in people with mild cognitive impairment (MCI). Cochrane Database Syst. Rev. 2015:CD010783. doi: 10.1002/14651858.CD010783.pub2, PMID: 25740785 PMC6464748

[ref4] Baumgart M. Snyder H. M. Carrillo M. C. Fazio S. Kim H. Johns H. (2015). Summary of the evidence on modifiable risk factors for cognitive decline and dementia: a population-based perspective. Alzheimers Dement. 11, 718–726. doi: 10.1016/j.jalz.2015.05.016, PMID: 26045020

[ref5] Bull F. C. Al-Ansari S. S. Biddle S. Borodulin K. Buman M. P. World Health Organization (2020). Guidelines on physical activity and sedentary behaviour. Br. J. Sports Med. 54, 1451–1462. doi: 10.1136/bjsports-2020-102955, PMID: 33239350 PMC7719906

[ref6] Buxton R. B. (2021). The thermodynamics of thinking: connections between neural activity, energy metabolism and blood flow. Philos. Trans. R. Soc. London B 376:20190624. doi: 10.1098/rstb.2019.0624, PMID: 33190604 PMC7741033

[ref7] Cabral H. (2008). Multiple comparisons procedures. Circulation 117, 698–701. doi: 10.1161/CIRCULATIONAHA.107.700971 18250280

[ref8] Cai H. Li G. Hua S. Liu Y. Chen L. (2017). Effect of exercise on cognitive function in chronic disease patients: a meta-analysis and systematic review of randomized controlled trials. Clin. Interv. Aging 12, 773–783. doi: 10.2147/CIA.S135700, PMID: 28546744 PMC5436795

[ref9] Cha J. (2022). Delivering personalized recommendations to support caregivers of people living with dementia: mixed methods study. JMIR Formativ. Res. 5:e35847. doi: 10.2196/35847 PMC9067568 35503650

[ref10] Chen Z. C. Wu H. Wang X. D. Zeng Y. Huang G. Lv Y. (2022). Association between marital status and cognitive impairment based on a cross-sectional study in China. Int. J. Geriatr. Psychiatry 37:5649. doi: 10.1002/gps.5649, PMID: 34729814

[ref11] Chen P. Yu E. S. Zhang M. Liu W. T. Hill R. Katzman R. (1995). ADL dependence and medical conditions in Chinese older persons: a population-based survey in Shanghai, China. J. Am. Geriatr. Soc. 43, 378–383. doi: 10.1111/j.1532-5415.1995.tb05811.x, PMID: 7706627

[ref12] Claassen J. Thijssen D. H. J. Panerai R. B. Faraci F. M. (2021). Regulation of cerebral blood flow in humans: physiology and clinical implications of autoregulation. Physiol. Rev. 101, 1487–1559. doi: 10.1152/physrev.00022.2020, PMID: 33769101 PMC8576366

[ref13] Ding D. Mutrie N. Bauman A. Pratt M. Hallal P. Powell K. (2020). Comprehensive and inclusive recommendations to activate populations. Lancet 396, 1780–1782. doi: 10.1016/S0140-6736(20)32229-7, PMID: 33248019

[ref14] Eto F. Tanaka M. Chishima M. Igarashi M. Mizoguchi T. Wada H.. (1992). Comprehensive activities of daily living (ADL) index for the elderly. Japan. J. Geriatrics 29, 841–848. doi: 10.3143/geriatrics.29.841, PMID: 1491480

[ref15] Farì G. Lunetti P. (2021). The effect of physical exercise on cognitive impairment in neurodegenerative disease: from pathophysiology to clinical and rehabilitative aspects. Int. J. Mol. Sci. 22:e632. doi: 10.3390/ijms222111632, PMID: 34769062 PMC8583932

[ref16] Gallardo-Gómez D. Del Pozo-Cruz J. Noetel M. Álvarez-Barbosa F. Alfonso-Rosa R. M. Del Pozo Cruz B. (2022). Optimal dose and type of exercise to improve cognitive function in older adults: a systematic review and Bayesian model-based network meta-analysis of RCTs. Ageing Res. Rev. 76:101591. doi: 10.1016/j.arr.2022.101591, PMID: 35182742

[ref17] Gao Y. Jia Z. (2023). The effect of activity participation in middle-aged and older people on the trajectory of depression in later life: National Cohort Study. JMIR Public Health Surveill. 9:e44682. doi: 10.2196/44682, PMID: 36951932 PMC10131905

[ref18] He F. Lin J. Li F. Zhai Y. Zhang T. Gu X.. (2021). Physical work and exercise reduce the risk of cognitive impairment in older adults: a population-based longitudinal study. J. Consult. Clin. Psychol. 18, 638–645. doi: 10.2174/1567205018666211118100451 34792012

[ref19] Hu F. F. Cheng G. R. Liu D. Liu Q. Gan X. G. Li L.. (2022). Population-attributable fractions of risk factors for all-cause dementia in China rural and urban areas: a cross-sectional study. Am. J. Alzheimers Dis. Other Dement. 269, 3147–3158. doi: 10.1007/s00415-021-10886-y 34839456

[ref20] Hu T. Zhou F. J. Chang Y. F. Li Y. S. Liu G. C. Hong Y.. (2015). miR21 is associated with the cognitive improvement following voluntary running wheel exercise in TBI mice. J. Molecul Neurosci. 57, 114–122. doi: 10.1007/s12031-015-0584-8, PMID: 26018937

[ref21] Huang X. Zhao X. Cai Y. Wan Q. (2022). The cerebral changes induced by exercise interventions in people with mild cognitive impairment and Alzheimer’s disease: a systematic review. Arch. Gerontol. Geriatr. 98:104547. doi: 10.1016/j.archger.2021.104547, PMID: 34634494

[ref22] Huang X. Zhao X. Li B. Cai Y. Zhang S. Wan Q.. (2022). Comparative efficacy of various exercise interventions on cognitive function in patients with mild cognitive impairment or dementia: a systematic review and network meta-analysis. J. Sport Health Sci. 11, 212–223. doi: 10.1016/j.jshs.2021.05.003, PMID: 34004389 PMC9068743

[ref23] Huang X. Zhao X. Li B. Cai Y. Zhang S. Yu F.. (2021). Biomarkers for evaluating the effects of exercise interventions in patients with MCI or dementia: a systematic review and meta-analysis. Exp. Gerontol. 151:111424. doi: 10.1016/j.exger.2021.111424 34051283

[ref24] Jachim S. K. Sakamoto A. E. Zhang X. Pearsall V. M. Schafer M. J. LeBrasseur N. K. (2020). Harnessing the effects of endurance exercise to optimize cognitive health: fundamental insights from Dr. mark P. Mattson. Ageing Res. Rev. 64:101147. doi: 10.1016/j.arr.2020.101147, PMID: 32814127 PMC7710559

[ref25] Jedrziewski M. K. Ewbank D. C. Wang H. Trojanowski J. Q. (2010). Exercise and cognition: results from the National Long Term Care Survey. Alzheimers Dement. 6, 448–455. doi: 10.1016/j.jalz.2010.02.004, PMID: 21044775 PMC2983109

[ref26] Jia R. X. Liang J. H. Xu Y. Wang Y. Q. (2019). Effects of physical activity and exercise on the cognitive function of patients with Alzheimer disease: a meta-analysis. BMC Geriatr. 19:181. doi: 10.1186/s12877-019-1175-2, PMID: 31266451 PMC6604129

[ref27] Jia L. Quan M. Fu Y. Zhao T. Li Y. Wei C.. (2020). Dementia in China: epidemiology, clinical management, and research advances. Lancet. Neurol. 19, 81–92. doi: 10.1016/S1474-4422(19)30290-X, PMID: 31494009

[ref28] Komiyama T. Tanoue Y. Sudo M. Costello J. T. Uehara Y. Higaki Y.. (2020). Cognitive impairment during high-intensity exercise: influence of cerebral blood flow. Med. Sci. Sports Exerc. 52, 561–568. doi: 10.1249/MSS.0000000000002183, PMID: 31609297

[ref29] Kurtze N. Rangul V. Hustvedt B. E. Flanders W. D. (2008). Reliability and validity of self-reported physical activity in the Nord-Trøndelag health study: HUNT 1. Scand. J. Public Health 36, 52–61. doi: 10.1177/1403494807085373, PMID: 18426785

[ref30] Lamb S. E. Sheehan B. Atherton N. Nichols V. Collins H. Mistry D.. (2018). Dementia and physical activity (DAPA) trial of moderate to high intensity exercise training for people with dementia: randomised controlled trial. J. Sports Sci. 361:k1675. doi: 10.1136/bmj.k1675 PMC5953238 29769247

[ref31] Law C. K. Lam F. M. Chung R. C. Pang M. Y. (2020). Physical exercise attenuates cognitive decline and reduces behavioural problems in people with mild cognitive impairment and dementia: a systematic review. J. Physiother. 66, 9–18. doi: 10.1016/j.jphys.2019.11.014, PMID: 31843427

[ref32] Liang F. Sun F. He B. Wang J. (2022). Treadmill exercise promotes microglial β-amyloid clearance and prevents cognitive decline in APP/PS1 mice. Neuroscience 491, 122–133. doi: 10.1016/j.neuroscience.2022.03.043, PMID: 35398179

[ref33] Liu W. Zhang J. Wang Y. Li J. Chang J. Jia Q. (2022). Effect of physical exercise on cognitive function of Alzheimer's disease patients: a systematic review and meta-analysis of randomized controlled trial. Front. Psych. 13:927128. doi: 10.3389/fpsyt.2022.927128, PMID: 35782450 PMC9243422

[ref34] López-Ortiz S. Valenzuela P. L. Seisdedos M. M. Morales J. S. Vega T. Castillo-García A.. (2021). Exercise interventions in Alzheimer’s disease: a systematic review and meta-analysis of randomized controlled trials. Ageing Res. Rev. 72:101479. doi: 10.1016/j.arr.2021.101479 34601135

[ref35] Madhu L. Somayaji Y. Shetty A. (2022). Promise of irisin to attenuate cognitive dysfunction in aging and Alzheimer’s disease. Ageing Res. Rev. 78:101637. doi: 10.1016/j.arr.2022.101637, PMID: 35504553 PMC9844023

[ref36] McKeith I. G. Boeve B. F. Dickson D. W. Halliday G. Taylor J. P. Weintraub D.. (2017). Diagnosis and management of dementia with Lewy bodies: fourth consensus report of the DLB consortium. Neurology 89, 88–100. doi: 10.1212/WNL.0000000000004058, PMID: 28592453 PMC5496518

[ref37] McKhann G. M. Knopman D. S. Chertkow H. Hyman B. T. Jack C. R. Kawas C. H.. (2011). The diagnosis of dementia due to Alzheimer’s disease: recommendations from the National Institute on Aging-Alzheimer’s Association workgroups on diagnostic guidelines for Alzheimer’s disease. Alzheimers Dement. 7, 263–269. doi: 10.1016/j.jalz.2011.03.005, PMID: 21514250 PMC3312024

[ref38] Morris J. C. (1993). The clinical dementia rating (CDR): current version and scoring rules. Neurology 43, 2412–2414. doi: 10.1212/WNL.43.11.2412-a 8232972

[ref39] Nakanishi K. Sakakima H. Norimatsu K. Otsuka S. Takada S. Tani A.. (2021). Effect of low-intensity motor balance and coordination exercise on cognitive functions, hippocampal Aβ deposition, neuronal loss, neuroinflammation, and oxidative stress in a mouse model of Alzheimer’s disease. Exp. Neurol. 337:113590. doi: 10.1016/j.expneurol.2020.113590, PMID: 33388314

[ref40] Norton S. Matthews F. E. Barnes D. E. Yaffe K. Brayne C. (2014). Potential for primary prevention of Alzheimer’s disease: an analysis of population-based data. Lancet. Neurol. 13, 788–794. doi: 10.1016/S1474-4422(14)70136-X, PMID: 25030513

[ref41] Ohtomo R. Kinoshita K. Ohtomo G. Takase H. Hamanaka G. Washida K.. (2020). Treadmill exercise suppresses cognitive decline and increases white matter oligodendrocyte precursor cells in a mouse model of prolonged cerebral Hypoperfusion. Oxidative Med. Cell. Longev. 11, 496–502. doi: 10.1007/s12975-019-00734-7 PMC7298669 31606888

[ref42] Pietrelli A. Matković L. Vacotto M. Lopez-Costa J. J. Basso N. Brusco A. (2018). Aerobic exercise upregulates the BDNF-serotonin systems and improves the cognitive function in rats. J. Am. Geriatr. Soc. 155, 528–542. doi: 10.1016/j.nlm.2018.05.007 29800645

[ref43] Qin Z. Han X. Ran J. Guo S. Lv L. (2022). Exercise-mediated alteration of miR-192-5p is associated with cognitive improvement in Alzheimer's disease. Neuroimmunomodulation 29, 36–43. doi: 10.1159/000516928, PMID: 34256371

[ref44] Román G. C. Tatemichi T. K. Erkinjuntti T. Cummings J. L. Masdeu J. C. Garcia J. H.. (1993). Vascular dementia: diagnostic criteria for research studies. Neurology 43, 250–260. PMID: 8094895 10.1212/wnl.43.2.250

[ref45] Soshi T. Andersson M. Kawagoe T. Nishiguchi S. Yamada M. Otsuka Y., Prefrontal plasticity after a 3-month exercise intervention in older adults relates to enhanced cognitive performance. Cerebral cortex New York, N.Y: (1991) 31, 4501–4517 10.1093/cercor/bhab102 34009242

[ref46] Steichele K. Keefer A. Dietzel N. Graessel E. Prokosch H. U. Kolominsky-Rabas P. L. (2022). The effects of exercise programs on cognition, activities of daily living, and neuropsychiatric symptoms in community-dwelling people with dementia-a systematic review. Alzheimers Res. Ther. 14:97. doi: 10.1186/s13195-022-01040-5, PMID: 35869496 PMC9306176

[ref47] Stillman C. M. Esteban-Cornejo I. Brown B. Bender C. M. Erickson K. I. (2020). Effects of exercise on brain and cognition across age groups and health states. Trends Neurosci. 43, 533–543. doi: 10.1016/j.tins.2020.04.010, PMID: 32409017 PMC9068803

[ref48] Tarassova O. Ekblom M. M. Moberg M. Lövdén M. Nilsson J. (2020). Peripheral BDNF response to physical and cognitive exercise and its association with cardiorespiratory fitness in healthy older adults. Front. Physiol. 11:1080. doi: 10.3389/fphys.2020.01080, PMID: 32982796 PMC7477111

[ref49] Triposkiadis F. Xanthopoulos A. (2023). Aortic stiffness: a major risk factor for multimorbidity in the elderly. J. Clin. Med. 12:2321. doi: 10.3390/jcm12062321, PMID: 36983321 PMC10058400

[ref50] Verges S. Rupp T. Jubeau M. Wuyam B. Esteve F. Levy P.. (2012). Cerebral perturbations during exercise in hypoxia. Am. J. Physiol. Regul. Integr. Comp. Physiol. 302, R903–R916. doi: 10.1152/ajpregu.00555.2011, PMID: 22319046

[ref51] Wang R. Holsinger R. M. D. (2018). Exercise-induced brain-derived neurotrophic factor expression: therapeutic implications for Alzheimer's dementia. Ageing Res. Rev. 48, 109–121. doi: 10.1016/j.arr.2018.10.002, PMID: 30326283

[ref52] Wang X. Zhu Y. T. Zhu Y. Sun Y. L. Huang J. Li Z.. (2022). Long-term running exercise alleviates cognitive dysfunction in APP/PSEN1 transgenic mice via enhancing brain lysosomal function. Acta Pharmacol. Sin. 43, 850–861. doi: 10.1038/s41401-021-00720-6, PMID: 34272505 PMC8976063

[ref53] Wen C. Hu H. Ou Y. Bi Y. Ma Y. Tan L.. (2021). Risk factors for subjective cognitive decline: the CABLE study. Transl. Psychiatry 11:576. doi: 10.1038/s41398-021-01711-1, PMID: 34753917 PMC8578345

[ref54] Wheeler M. J. Green D. J. Ellis K. A. Cerin E. Heinonen I. Naylor L. H.. (2020). Distinct effects of acute exercise and breaks in sitting on working memory and executive function in older adults: a three-arm, randomised cross-over trial to evaluate the effects of exercise with and without breaks in sitting on cognition. Br. J. Sports Med. 54, 776–781. doi: 10.1136/bjsports-2018-100168, PMID: 31036563

[ref55] Xu W. Tan L. Su B. Yu H. Bi Y. Yue X.. (2020). Sleep characteristics and cerebrospinal fluid biomarkers of Alzheimer's disease pathology in cognitively intact older adults: the CABLE study. Alzheimers Dement. 16, 1146–1152. doi: 10.1002/alz.12117, PMID: 32657026

[ref56] Yamada Y. Frith E. M. Wong V. Spitz R. W. Bell Z. W. Chatakondi R. N.. (2021). Acute exercise and cognition: a review with testable questions for future research into cognitive enhancement with blood flow restriction. Med. Hypotheses 151:110586. doi: 10.1016/j.mehy.2021.110586, PMID: 33848917

[ref57] Zhang X. He Q. Huang T. Zhao N. Liang F. Xu B.. (2019). Treadmill exercise decreases Aβ deposition and counteracts cognitive decline in APP/PS1 mice, possibly via hippocampal microglia modifications. Front. Aging Neurosci. 11:78. doi: 10.3389/fnagi.2019.00078, PMID: 31024293 PMC6461026

[ref58] Zhong S. Zhao B. Ma Y. Sun Y. Zhao Y. Liu W.. (2022). Associations of physical activity with Alzheimer’s disease pathologies and cognition: the CABLE study. J. Alzheimer’s Disease 89, 483–492. doi: 10.3233/JAD-220389, PMID: 35871345

[ref59] Zotcheva E. Håberg A. K. Wisløff U. Salvesen Ø. Selbæk G. Stensvold D.. (2022). Effects of 5 years aerobic exercise on cognition in older adults: the generation 100 study: a randomized controlled trial. Sports Med. 52, 1689–1699. doi: 10.1007/s40279-021-01608-5, PMID: 34878637 PMC9213353

