# Supplementary material for: Physical exercise frequency and cognition: a multicenter cross-sectional cohort study
Source: Front Aging Neurosci. 2024 Mar 8;16:1381692. doi: 10.3389/fnagi.2024.1381692 (PMC10958531; doi:10.3389/fnagi.2024.1381692)
Supplement: Supplementary file 1 [file Data_Sheet_1.docx]

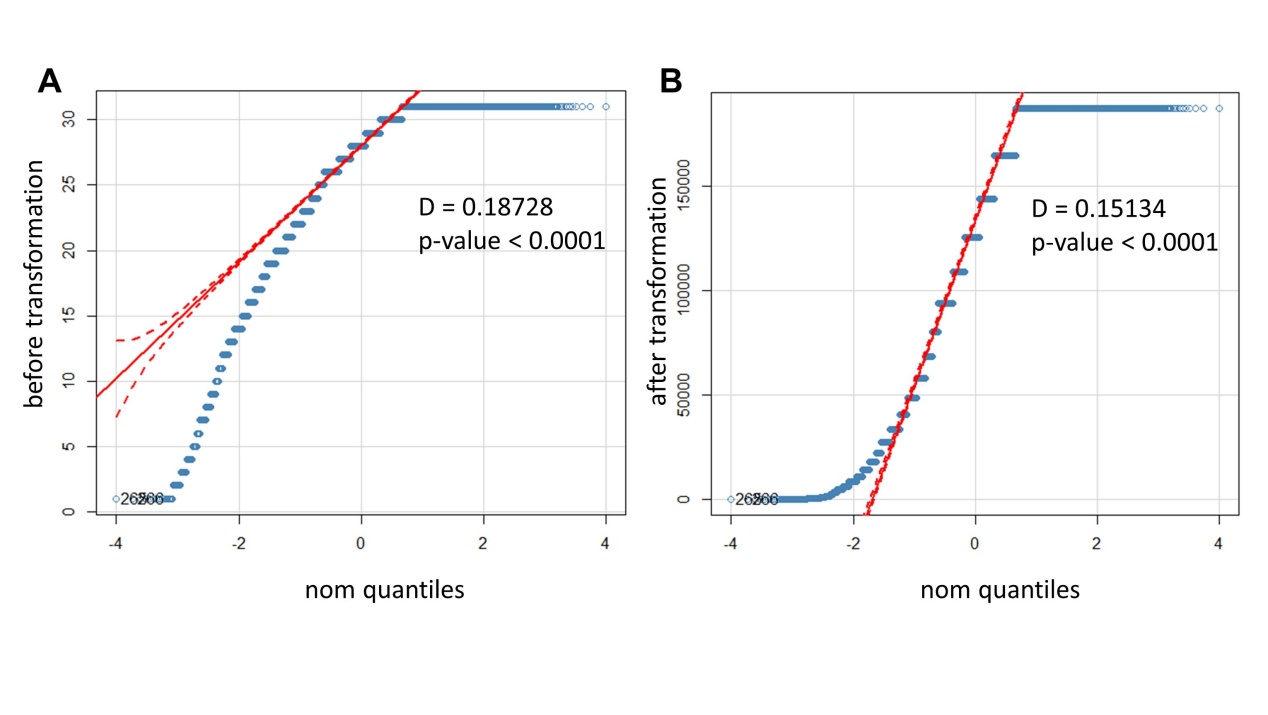
**eFigure 1.** Quantile-Quantile plots

**eTable 1.Univariate logistic regression analyses for relationship between physical exercise and dementia**

|  | **OR** | **LCI** | **UCI** | **p value** |  |
| --- | --- | --- | --- | --- | --- |
| Age (years) | 1.0991 | 1.0908 | 1.1074 | <0.0001 | |
| Sex (female) | 1.3474 | 1.2129 | 1.4981 | <0.0001 | |
| Education (years) | 0.9416 | 0.9312 | 0.9312 | <0.0001 | |
| Hypertension (yes) | 1.0032 | 0.9056 | 1.1113 | 0.9520 | |
| DM (yes) | 0.9714 | 0.8389 | 1.1203 | 0.6940 | |
| CHD (yes) | 1.0553 | 0.9135 | 1.2145 | 0.4590 | |
| Stroke (yes) | 1.4401 | 1.2565 | 1.6457 | <0.0001 | |
| Smoking (yes) | 1.0876 | 0.9690 | 1.2188 | 0.1510 | |
| Drinking (yes) | 1.0027 | 0.8871 | 1.1309 | 0.9660 | |
| Physical exercise | 0.4212 | 0.3569 | 0.4994 | <0.0001 | |
| Frequency of physical exercise (ref: Never) | |  |  |  | |
| 1-2 times/week | 0.7336 | 0.5838 | 0.9212 | 0.0077 | |
| 3 times/week | 0.5591 | 0.4531 | 0.6906 | <0.0001 | |
| 4-5 times/week | 0.3727 | 0.3118 | 0.4470 | <0.0001 | |
| ＞5 times/week | 0.3778 | 0.3142 | 0.4556 | <0.0001 | |

**Abbreviations:** DM, diabetes mellitus; CHD, coronary heart disease.

**eTable 2. Univariate linear regression analyses for relationship between physical exercise and dementia**

|  | **β** | **LCI** | **UCI** | **p value** |  |
| --- | --- | --- | --- | --- | --- |
| Age (years) | 26944 | 23250 | 30638 | <0.0001 | |
| Sex (female) | -2476 | -2610 | -2343 | <0.0001 | |
| Education (years) | -18882 | -20624 | -17140 | <0.0001 | |
| Hypertension (yes) | -1880 | -3631 | -128 | 0.0355 | |
| DM (yes) | 507 | -1948 | 2962 | 0.6850 | |
| CHD (yes) | -2594 | -5067 | -121 | 0.0398 | |
| Stroke (yes) | -7548 | -10088 | -5009 | <0.0001 | |
| Smoking (yes) | 2279 | 286 | 4272 | 0.0250 | |
| Drinking (yes) | 4878 | 2799 | 6956 | <0.0001 | |
| Physical exercise | 26944 | 23250 | 30638 | <0.0001 | |
| Frequency of physical exercise (ref: Never) | |  |  |  | |
| 1-2 times/week | 9259 | 4398 | 14119 | 0.0002 | |
| 3 times/week | 18463 | 14093 | 22834 | <0.0001 | |
| 4-5 times/week | 26858 | 23063 | 30654 | <0.0001 | |
| ＞5 times/week | 33721 | 29856 | 37586 | <0.0001 | |

**Abbreviations:** DM, diabetes mellitus; CHD, coronary heart disease.

**eTable 3. Logistic regression analyses for relationship between physical exercise and dementia**

|  | **OR** | **LCI** | **UCI** | **p value** |
| --- | --- | --- | --- | --- |
| **M1** |  |  |  |  |
| Physical exercise | 0.4212 | 0.3569 | 0.4994 | **<0.0001** |
| Frequency of physical exercise (ref: Never) | | | | |
| 1-2 times/week | 0.7336 | 0.5838 | 0.9212 | **0.0077** |
| 3 times/week | 0.5591 | 0.4531 | 0.6906 | **<0.0001** |
| 4-5 times/week | 0.3727 | 0.3118 | 0.4470 | **<0.0001** |
| ＞5 times/week | 0.3778 | 0.3142 | 0.4556 | **<0.0001** |
| **M2** |  |  |  |  |
| Physical exercise | 0.5379 | 0.4512 | 0.6441 | **<0.0001** |
| Frequency of physical exercise (ref: Never) | | | | |
| 1-2 times/week | 0.7927 | 0.6244 | 1.0059 | 0.0561 |
| 3 times/week | 0.6497 | 0.5217 | 0.8099 | **0.0001** |
| 4-5 times/week | 0.4772 | 0.3956 | 0.5778 | **<0.0001** |
| ＞5 times/week | 0.5044 | 0.4150 | 0.6150 | **<0.0001** |
| **M3** |  |  |  |  |
| Physical exercise | 0.5414 | 0.4536 | 0.6491 | **<0.0001** |
| Frequency of physical exercise (ref: Never) | | | | |
| 1-2 times/week | 0.7908 | 0.6222 | 1.0045 | 0.0546 |
| 3 times/week | 0.6619 | 0.5310 | 0.8259 | **0.0002** |
| 4-5 times/week | 0.4794 | 0.3969 | 0.5811 | **<0.0001** |
| ＞5 times/week | 0.5053 | 0.4152 | 0.6170 | **<0.0001** |

Abbreviations: OR, odds ratio; LCI, lower confidence interval (2.5%); UCI, upper confidence interval (97.5%).

M1: The univariate logistic regression analysis.

M2: The multivariate logistic regression analysis adjusted for age, sex and education years.

M3: The multivariate logistic regression analysis adjusted for age, sex and education years, hypertension, DM, CHD, stroke, smoking and drinking.

**eTable 4. Liner regression analyses for relationship between physical exercise and cognition**

|  | **M1** | | **M2** | | **M3** | |
| --- | --- | --- | --- | --- | --- | --- |
|  | **β** | **p value** | **β** | **p value** | **β** | **p value** |
| Physical exercise | 26944 | **<0.0001** | 13013 | **<0.0001** | 12851 | **<0.0001** |
| Frequency of physical exercise (ref: Never) | | |  |  |  |  |
| 1-2 times/week | 9259 | **0.0002** | 6675 | **0.0015** | 6788 | **0.0012** |
| 3 times/week | 18463 | **<0.0001** | 12823 | **<0.0001** | 12648 | **<0.0001** |
| 4-5 times/week | 26858 | **<0.0001** | 13395 | **<0.0001** | 13221 | **<0.0001** |
| ＞5 times/week | 33721 | **<0.0001** | 14222 | **<0.0001** | 14036 | **<0.0001** |

M1: The univariate liner regression analysis.

M2: The multivariate liner regression analysis adjusted for age, sex and education years.

M3: The multivariate liner regression analysis adjusted for age, sex and education years, hypertension, DM, CHD, stroke, smoking and drinking.

**eTable 5. Logistic regression analyses between physical exercise and dementia subtype**

|  | **AD** | | **VD** | | **Other types of dementia** | |
| --- | --- | --- | --- | --- | --- | --- |
|  | **OR** | **P** | **OR** | **P** | **OR** | **P** |
| **M1** |  |  |  |  |  |  |
| Physical exercise | 0.4873(0.3962-0.6046) | **<0.0001** | 0.3582(0.2501-0.5295) | **<0.0001** | 0.3145(0.2317-0.4359) | **<0.0001** |
| Frequency of physical exercise (ref: Never) | | | | | | |
| 1-2 times/week | 0.7974(0.6000-1.0594) | 0.1180 | 0.7454(0.4486-1.2340) | 0.1591 | 0.5808(0.3679-0.9072) | **0.0179** |
| 3 times/week | 0.5959(0.4577-0.7775) | **0.0001** | 0.5201(0.3228-0.8416) | **0.0180** | 0.5026(0.3363-0.7525) | **0.0008** |
| 4-5 times/week | 0.4294(0.3439-0.5400) | **<0.0001** | 0.3070(0.2059-0.4684) | **<0.0001** | 0.2890(0.2060-0.4114) | **<0.0001** |
| ＞5 times/week | 0.4664(0.3439-0.5893) | **<0.0001** | 0.2957(0.1939-0.4590) | **<0.0001** | 0.2328(0.1607-0.3402) | **<0.0001** |
| **M2** |  |  |  |  |  |  |
| Physical exercise | 0.5847(0.4712-0.7318) | **<0.0001** | 0.4211(0.2920-0.6262) | **<0.0001** | 0.4610(0.3338-0.6495) | **<0.0001** |
| Frequency of physical exercise (ref: Never) | | | | | | |
| 1-2 times/week | 0.8627(0.5676-1.1558) | 0.3220 | 0.7845(0.4703-1.3042) | 0.3488 | 0.6035(0.3759-0.9595) | **0.0341** |
| 3 times/week | 0.6768(0.5161-0.8898) | **0.0049** | 0.5801(0.3586-0.9424) | **0.0265** | 0.6028(0.3972-0.9170) | **0.0174** |
| 4-5 times/week | 0.5170(0.4107-0.6556) | **<0.0001** | 0.3568(0.2378-0.5475) | **<0.0001** | 0.4256(0.2985-0.6156) | **<0.0001** |
| ＞5 times/week | 0.5676(0.4477-0.6556) | **<0.0001** | 0.3531(0.2294-0.5529) | **<0.0001** | 0.3933(0.2666-0.5850) | **<0.0001** |
| **M3** |  |  |  |  |  |  |
| Physical exercise | 0.5708(0.4578-0.7178) | **<0.0001** | 0.3864(0.2536-0.6052) | **<0.0001** | 0.4749(0.2501-0.5295) | **<0.0001** |
| Frequency of physical exercise (ref: Never) | | | | | | |
| 1-2 times/week | 0.8423(0.6249-1.1352) | 0.2593 | 0.6638(0.3741-1.1751) | 0.1591 | 0.6601(0.4064-1.0625) | 0.0893 |
| 3 times/week | 0.6741(0.5115-0.8906) | **0.0053** | 0.5216(0.3044-0.8981) | **0.0180** | 0.6148(0.4004-0.9462) | **0.0262** |
| 4-5 times/week | 0.5055(0.3996-0.6439) | **<0.0001** | 0.3328(0.2101-0.5383) | **<0.0001** | 0.4361(0.3029-0.6369) | **<0.0001** |
| ＞5 times/week | 0.5450(0.4276-0.6992) | **<0.0001** | 0.3239(0.1994-0.5346) | **<0.0001** | 0.4033(0.2707-0.6057) | **<0.0001** |

Abbreviations: OR, odds ratio; LCI, lower confidence interval (2.5%); UCI, upper confidence interval (97.5%).

M1: The univariate logistic regression analysis.

M2: The multivariate logistic regression analysis adjusted for age, sex and education years.

M3: The multivariate logistic regression analysis adjusted for age, sex and education years, hypertension, DM, CHD, stroke, smoking and drinking.

**eTable 6. Liner regression analyses for relationship between physical exercise and cognition in dementia subtype**

|  | **AD** | | **VD** | | **Other types of dementia** | |
| --- | --- | --- | --- | --- | --- | --- |
|  | **β** | **p value** | **β** | **p value** | **β** | **p value** |
| **M1** |  |  |  |  |  |  |
| Physical exercise | 22485 | **<0.0001** | 19169 | **<0.0001** | 20635 | **<0.0001** |
| Frequency of physical exercise (ref: Never) | | |  |  |  |  |
| 1-2 times/week | 7142 | **0.0038** | 6247 | **0.0090** | 7519 | **0.0018** |
| 3 times/week | 15570 | **<0.0001** | 13000 | **<0.0001** | 13988 | **<0.0001** |
| 4-5 times/week | 22039 | **<0.0001** | 18010 | **<0.0001** | 19404 | **<0.0001** |
| ＞5 times/week | 28526 | **<0.0001** | 25323 | **<0.0001** | 27111 | **<0.0001** |
| **M2** |  |  |  |  |  |  |
| Physical exercise | 10460 | **<0.0001** | 9093 | **<0.0001** | 9234 | **<0.0001** |
| Frequency of physical exercise (ref: Never) | | | |  |  |  |
| 1-2 times/week | 5167 | **0.0140** | 5691 | **0.0044** | 6491 | **0.0011** |
| 3 times/week | 11262 | **<0.0001** | 10186 | **<0.0001** | 10573 | **<0.0001** |
| 4-5 times/week | 10555 | **<0.0001** | 8640 | **<0.0001** | 8705 | **<0.0001** |
| ＞5 times/week | 11312 | **<0.0001** | 10099 | **<0.0001** | 10102 | **<0.0001** |
| **M3** |  |  |  |  |  |  |
| Physical exercise | 10406 | **<0.0001** | 8806 | **<0.0001** | 9043 | **<0.0001** |
| Frequency of physical exercise (ref: Never) | | | |  |  |  |
| 1-2 times/week | 5219 | **0.0130** | 5907 | **0.0030** | 6407 | **0.0012** |
| 3 times/week | 11134 | **<0.0001** | 9987 | **<0.0001** | 10458 | **<0.0001** |
| 4-5 times/week | 10464 | **<0.0001** | 8334 | **<0.0001** | 8525 | **<0.0001** |
| ＞5 times/week | 11325 | **<0.0001** | 9694 | **<0.0001** | 9839 | **<0.0001** |

M1: The univariate liner regression analysis.

M2: The multivariate liner regression analysis adjusted for age, sex and education years.

M3: The multivariate liner regression analysis adjusted for age, sex and education years, hypertension, DM, CHD, stroke, smoking and drinking.

**eTable 7. Evaluation of multicollinearity in linear regression analyses**

| **VIF** | **Total** | **AD** | **VD** | **Other dementia**  **types** |
| --- | --- | --- | --- | --- |
| Physical exercise | 1.01 | 1.01 | 1.01 | 1.01 |
| Age (years) | 1.02 | 1.01 | 1.01 | 1.01 |
| Sex (female) | 1.16 | 1.16 | 1.17 | 1.17 |
| Education (years) | 1.07 | 1.07 | 1.07 | 1.07 |
| Hypertension | 1.06 | 1.05 | 1.06 | 1.05 |
| DM | 1.03 | 1.03 | 1.03 | 1.03 |
| CHD | 1.04 | 1.04 | 1.05 | 1.04 |
| Stroke | 1.03 | 1.03 | 1.04 | 1.03 |
| Smoking | 1.42 | 1.42 | 1.44 | 1.43 |
| Drinking | 1.41 | 1.41 | 1.42 | 1.41 |
| Physical exercise frequency | 1.06 | 1.05 | 1.05 | 1.05 |

Abbreviations: VIF, variance inflation factor; AD, Alzheimer's dementia; VD, vascular dementia; DM, diabetes mellitus; CHD, coronary heart disease.

**eTable 8. Interaction analyses between physical exercise and dementia**

| **p value** | **Physical exercise** | **Frequency of physical exercise (ref: Never, times/week)** | | | |
| --- | --- | --- | --- | --- | --- |
|  |  | **1-2** | **3** | **4-5** | **＞5** |
| **Age (≥75)** | **0.03** | 0.43 | 0.44 | **0.02** | **0.02** |
| Sex (female) | 0.84 | 0.49 | 0.63 | 0.85 | 0.90 |
| **Hypertension** | **0.04** | 0.12 | 0.44 | 0.13 | **0.01** |
| DM | 0.34 | 0.71 | 0.12 | 0.51 | 0.36 |
| CHD | 0.28 | 0.11 | 0.69 | 0.40 | 0.43 |
| **Stroke** | **<0.01** | 0.10 | 0.18 | **<0.01** | **<0.01** |
| Smoking | 0.15 | 0.83 | 0.95 | 0.09 | 0.06 |
| Drinking | 0.32 | 0.81 | 0.94 | 0.31 | 0.08 |

Abbreviations: DM, diabetes mellitus; CHD, coronary heart disease.

**eTable 9. Subgroup analyses stratified by age**

|  | **65-74** | | | | **≥75** | | | |
| --- | --- | --- | --- | --- | --- | --- | --- | --- |
|  | OR | LCI | UCI | p value | OR | LCI | UCI | p value |
| **M1** |  |  |  |  |  |  |  |  |
| Physical exercise | 0.57 | 0.42 | 0.79 | **<0.01** | 0.38 | 0.31 | 0.47 | **<0.01** |
| Frequency of physical exercise (ref: Never) | |  |  |  |  |  |  |  |
| **1-2 times/week** | 0.85 | 0.56 | 1.29 | 0.44 | 0.69 | 0.52 | 0.92 | **0.01** |
| 3 times/week | 0.65 | 0.45 | 0.96 | **0.03** | 0.55 | 0.42 | 0.71 | **<0.01** |
| 4-5 times/week | 0.53 | 0.39 | 0.74 | **<0.01** | 0.33 | 0.26 | 0.41 | **<0.01** |
| ＞5 times/week | 0.55 | 0.39 | 0.77 | **<0.01** | 0.33 | 0.26 | 0.42 | **<0.01** |
| **M2** |  |  |  |  |  |  |  |  |
| Physical exercise | 0.58 | 0.43 | 0.80 | **<0.01** | 0.52 | 0.42 | 0.65 | **<0.01** |
| Frequency of physical exercise (ref: Never) | | |  |  |  |  |  |  |
| 1-2 times/week | 0.84 | 0.55 | 1.27 | 0.41 | 0.77 | 0.58 | 1.04 | 0.09 |
| 3 times/week | 0.66 | 0.45 | 0.98 | **0.03** | 0.65 | 0.49 | 0.85 | **<0.01** |
| 4-5 times/week | 0.53 | 0.38 | 0.74 | **<0.01** | 0.45 | 0.36 | 0.58 | **<0.01** |
| ＞5 times/week | 0.56 | 0.40 | 0.79 | **<0.01** | 0.48 | 0.38 | 0.61 | **<0.01** |
| **M3** |  |  |  |  |  |  |  |  |
| Physical exercise | 0.57 | 0.42 | 0.79 | **<0.01** | 0.53 | 0.42 | 0.66 | **<0.01** |
| Frequency of physical exercise (ref: Never) | |  |  |  |  |  |  |  |
| 1-2 times/week | 0.81 | 0.53 | 1.23 | 0.32 | 0.78 | 0.58 | 1.04 | 0.09 |
| 3 times/week | 0.65 | 0.45 | 0.96 | **0.03** | 0.66 | 0.51 | 0.87 | **<0.01** |
| 4-5 times/week | 0.52 | 0.38 | 0.73 | **<0.01** | 0.45 | 0.36 | 0.58 | **<0.01** |
| ＞5 times/week | 0.55 | 0.39 | 0.78 | **<0.01** | 0.48 | 0.37 | 0.61 | **<0.01** |

Abbreviations: OR, odds ratio; LCI, lower confidence interval (2.5%); UCI, upper confidence interval (97.5%).

M1: The univariate logistic regression analysis.

M2: The multivariate logistic regression analysis adjusted for age, sex and education years.

M3: The multivariate logistic regression analysis adjusted for age, sex and education years, hypertension, DM, CHD, stroke, smoking and drinking.

**eTable 10. Subgroup analyses stratified by stroke**

|  | **Without stroke** | | | | **Stroke** | | | |
| --- | --- | --- | --- | --- | --- | --- | --- | --- |
|  | OR | LCI | UCI | p value | OR | LCI | UCI | p value |
| **M1** |  |  |  |  |  |  |  |  |
| Physical exercise | 0.50 | 0.41 | 0.60 | **<0.01** | 0.25 | 0.17 | 0.35 | **<0.01** |
| Frequency of physical exercise (ref: Never) | |  |  |  |  |  |  |  |
| **1-2 times/week** | 0.81 | 0.62 | 1.05 | 0.12 | 0.52 | 0.32 | 0.83 | **0.01** |
| 3 times/week | 0.61 | 0.48 | 0.78 | **<0.01** | 0.43 | 0.28 | 0.68 | **<0.01** |
| 4-5 times/week | 0.45 | 0.36 | 0.55 | **<0.01** | 0.20 | 0.13 | 0.29 | **<0.01** |
| ＞5 times/week | 0.46 | 0.37 | 0.57 | **<0.01** | 0.18 | 0.12 | 0.27 | **<0.01** |
| **M2** |  |  |  |  |  |  |  |  |
| Physical exercise | 0.62 | 0.50 | 0.76 | **<0.01** | 0.34 | 0.24 | 0.50 | **<0.01** |
| Frequency of physical exercise (ref: Never) | | |  |  |  |  |  |  |
| 1-2 times/week | 0.84 | 0.63 | 1.10 | 0.20 | 0.67 | 0.41 | 1.09 | 0.11 |
| 3 times/week | 0.69 | 0.54 | 0.89 | **<0.01** | 0.57 | 0.36 | 0.91 | **0.02** |
| 4-5 times/week | 0.56 | 0.45 | 0.70 | **<0.01** | 0.27 | 0.18 | 0.41 | **<0.01** |
| ＞5 times/week | 0.61 | 0.49 | 0.76 | **<0.01** | 0.25 | 0.16 | 0.39 | **<0.01** |
| **M3** |  |  |  |  |  |  |  |  |
| Physical exercise | 0.61 | 0.50 | 0.75 | **<0.01** | 0.34 | 0.23 | 0.49 | **<0.01** |
| Frequency of physical exercise (ref: Never) | |  |  |  |  |  |  |  |
| 1-2 times/week | 0.83 | 0.63 | 1.09 | 0.18 | 0.64 | 0.39 | 1.05 | 0.08 |
| 3 times/week | 0.69 | 0.53 | 0.89 | **<0.01** | 0.56 | 0.35 | 0.89 | **0.02** |
| 4-5 times/week | 0.55 | 0.44 | 0.69 | **<0.01** | 0.27 | 0.18 | 0.41 | **<0.01** |
| ＞5 times/week | 0.59 | 0.47 | 0.75 | **<0.01** | 0.25 | 0.16 | 0.38 | **<0.01** |

Abbreviations: OR, odds ratio; LCI, lower confidence interval (2.5%); UCI, upper confidence interval (97.5%).

M1: The univariate logistic regression analysis.

M2: The multivariate logistic regression analysis adjusted for age, sex and education years.

M3: The multivariate logistic regression analysis adjusted for age, sex and education years, hypertension, DM, CHD, stroke, smoking and drinking.

**eTable 11. Subgroup analyses stratified by hypertension**

|  | **Without hypertension** | | | | **Hypertension** | | | |
| --- | --- | --- | --- | --- | --- | --- | --- | --- |
|  | OR | LCI | UCI | p value | OR | LCI | UCI | p value |
| M1 |  |  |  |  |  |  |  |  |
| Physical exercise | 0.35 | 0.28 | 0.45 | **<0.01** | 0.50 | 0.39 | 0.64 | **<0.01** |
| Frequency of physical exercise (ref: Never) | |  |  |  |  |  |  |  |
| **1-2 times/week** | 0.61 | 0.44 | 0.84 | **<0.01** | 0.88 | 0.64 | 1.21 | 0.43 |
| 3 times/week | 0.51 | 0.38 | 0.69 | **<0.01** | 0.61 | 0.45 | 0.82 | **<0.01** |
| 4-5 times/week | 0.32 | 0.25 | 0.42 | **<0.01** | 0.43 | 0.33 | 0.56 | **<0.01** |
| ＞5 times/week | 0.29 | 0.23 | 0.38 | **<0.01** | 0.48 | 0.37 | 0.63 | **<0.01** |
| M2 |  |  |  |  |  |  |  |  |
| Physical exercise | 0.46 | 0.36 | 0.60 | **<0.01** | 0.62 | 0.48 | 0.80 | **<0.01** |
| Frequency of physical exercise (ref: Never) | | |  |  |  |  |  |  |
| **1-2 times/week** | 0.63 | 0.44 | 0.88 | **0.01** | 1.00 | 0.71 | 1.40 | 0.99 |
| 3 times/week | 0.60 | 0.44 | 0.82 | **<0.01** | 0.70 | 0.51 | 0.96 | **0.03** |
| 4-5 times/week | 0.42 | 0.33 | 0.56 | **<0.01** | 0.53 | 0.41 | 0.70 | **<0.01** |
| ＞5 times/week | 0.41 | 0.31 | 0.54 | **<0.01** | 0.61 | 0.47 | 0.81 | **<0.01** |
| M3 |  |  |  |  |  |  |  |  |
| Physical exercise | 0.46 | 0.36 | 0.60 | **<0.01** | 0.63 | 0.49 | 0.81 | **<0.01** |
| Frequency of physical exercise (ref: Never) | |  |  |  |  |  |  |  |
| **1-2 times/week** | 0.62 | 0.44 | 0.87 | **0.01** | 1.00 | 0.71 | 1.40 | 0.99 |
| 3 times/week | 0.60 | 0.44 | 0.83 | **<0.01** | 0.71 | 0.52 | 0.98 | **0.03** |
| 4-5 times/week | 0.42 | 0.32 | 0.55 | **<0.01** | 0.54 | 0.41 | 0.71 | **<0.01** |
| ＞5 times/week | 0.40 | 0.31 | 0.54 | **<0.01** | 0.62 | 0.47 | 0.82 | **<0.01** |

Abbreviations: OR, odds ratio; LCI, lower confidence interval (2.5%); UCI, upper confidence interval (97.5%).

M1: The univariate logistic regression analysis.

M2: The multivariate logistic regression analysis adjusted for age, sex and education years.

M3: The multivariate logistic regression analysis adjusted for age, sex and education years, hypertension, DM, CHD, stroke, smoking and drinking.
